# Adaptation to monogamy influences parental care but not mating behavior in the burying beetle, *Nicrophorus vespilloides*

**DOI:** 10.1002/ece3.6387

**Published:** 2020-05-22

**Authors:** Matthew Schrader, Madolin K. Keller, Garrett F. Lucey

**Affiliations:** ^1^ Department of Biology University of the South Sewanee Tennessee USA

**Keywords:** mating system, *Nicrophorus vespilloides*, parental care

## Abstract

The mating system is expected to have an important influence on the evolution of mating and parenting behaviors. Although many studies have used experimental evolution to examine how mating behaviors evolve under different mating systems, this approach has seldom been used to study the evolution of parental care. We used experimental evolution to test whether adaptation to different mating systems involves changes in mating and parenting behaviors in populations of the burying beetle, *Nicrophorus vespilloides*. We maintained populations under monogamy or promiscuity for six generations. This manipulation had an immediate impact on reproductive performance and adult survival. Compared to monogamy, promiscuity reduced brood size and adult (particularly male) survival during breeding. After six generations of experimental evolution, there was no divergence between monogamous and promiscuous populations in mating behaviors. Parents from the promiscuous populations (especially males) displayed less care than parents from the monogamous populations. Our results are consistent with the hypothesis that male care will increase with the certainty of paternity. However, it appears that this change is not associated with a concurrent change in mating behaviors.

## Graphical abstract

We used experimental evolution to test whether adaptation to monogamy involved changes in mating and parenting behaviors in the burying beetle, *Nicrophorus vespilloides*. We found that males from monogamous and promiscuous populations did not differ in mating behaviors. However, males from monogamous populations were more attentive parents than males from promiscuous populations.

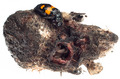

## 1 INTRODUCTION

Parental care has evolved repeatedly across the animal kingdom and the means by which parents care for their young are incredibly diverse (Clutton‐Brock, 1991; Smiseth, Kölliker, & Royle, 2012). A major goal of behavioral ecology has been to understand this diversity, and there is now a considerable body of work focused on the factors that explain why parental care evolves (Klug, Alonzo, & Bonsall, 2012; Klug & Bonsall, 2010), which sex is the predominate caregiver (Henshaw, Fromhage, & Jones, 2019; Székely & Reynolds, 1995), why species vary in the amount or duration of care they provide (Capodeanu‐Nägler et al., 2016), and how conflicts over care are manifested and resolved (Kilner & Hinde, 2012; Trivers, 1974). The mating system is expected to play an important role in shaping much of this diversity because it is likely to influence the costs and benefits of parental care for both sexes (Parker, Royle, & Hartley, 2002; Trivers, 1974).

The impact of the mating system on the evolution of male care has attracted particular attention for two reasons. First, the mating system will usually have a greater impact on certainty of paternity than certainty of maternity. Several models have asked whether males should facultatively adjust the level of care that they provide in response to their certainty of paternity (Westneat & Sargent, 1996). Early models predicted that male care should be insensitive to certainty of paternity (Grafen, 1980; Maynard Smith, 1978). However, more recent models predict that males should facultatively increase the level of care that they provide as their certainty of paternity increases, so long as there are reliable paternity cues and male investment is costly (Westneat & Sargent, 1996; Westneat & Sherman, 1993). Nonfacultative adjustments of paternal care in response to certainty of paternity are also predicted by theory, with selection favoring lower levels of male care in populations where certainty of paternity is on average low and higher levels of care in populations where certainty of paternity is on average high (Westneat & Sherman, 1993). This relationship is predicted even in the absence of paternity cues, as long as the expression of male care is costly (Westneat & Sargent, 1996; Westneat & Sherman, 1993).

Behavioral correlations between male–male competition and parental care provide a second potential link between the mating system and the expression of male parental care. For example, in some species the amount of aggression that a male displays in the context of male–male competition is negatively correlated with the amount of care that he provides (e.g., Duckworth, 2006; Hunt & Simmons, 2002; McGlothlin, Jawor, & Ketterson, 2007). These correlations suggest that selection in mating systems characterized by intense male–male competition (where aggression may be important to the outcome of male–male competition) might result in a correlated decline in male care.

Although there are reasons to suspect that the mating system will influence the evolution male parental care, direct links between aspects of the mating system and male care have been elusive. For example, empirical support for a positive relationship between certainty of paternity and paternal care is mixed (Alonzo, 2010): some studies have found this predicted relationship (Disciullo, Thompson, & Sakaluk, 2019; Hunt & Simmons, 2002; Sheldon & Ellegren, 1998), others have found either no relationship between certainty of paternity and paternal care (Sakaluk & Müller, 2008), or a negative relationship between the two (Alonzo & Heckman, 2010; Hopwood, Moore, Tregenza, & Royle, 2015). There are two major explanations for the lack of a consistent relationship between multiple paternity and male care. First, the prediction that males will facultatively reduce the amount of care they provide with decreased certainty of paternity assumes that males have information regarding their certainty of paternity (i.e., there are paternity cues) and that there is a trade‐off between male parental investment in the current brood and investment in future broods (Westneat & Sargent, 1996). If these assumptions are not met, then facultative adjustment of male care in response to paternity cues may be impossible or inconsequential for male fitness. Second, Alonzo (2010) has suggested that theory linking multiple paternity to reduced male care has ignored the potential for social and coevolutionary feedbacks between mating and parenting behaviors. According to this view, mating and parenting behaviors expressed by both sexes are potentially linked via phenotypic trade‐offs (within and between the sexes), social plasticity, and genetic correlations (within and between the sexes). Understanding how one aspect of this network will evolve in response to a change in the mating system must consider the network of potentially interacting traits (Alonzo, 2010; Head, Hinde, Moore, & Royle, 2014; Royle, Alonzo, & Moore, 2016).

The social and coevolutionary dynamics described by Alonzo (2010) are likely to be important in many animals. However, incorporating this complexity into new theory and empirical work is a major challenge. On the empirical front, Alonzo (2010) and Royle et al. (2016) have argued that studies employing artificial selection and experimental evolution (Kawecki et al., 2012) are likely to further our understanding of coevolutionary dynamics between mating and parenting in at least two ways. First, artificial selection experiments can reveal genetic correlations between traits that are decoupled in their expression (such as mating and parenting behaviors) but that may represent concerted responses to selection. Head et al. (2014) provide an excellent example of an artificial selection study in which selection on a paternity assurance trait (male repeated mating rate) resulted in a correlated response in parental care behaviors in the burying beetle *Nicrophorus vespilloides*. They found that selection for increased male mating rate led to a correlated decline in female parental care, but no change in male care. This result suggests that the evolution of a paternity assurance trait expressed in males can have an impact on the expression of parental care behaviors expressed in females, a pattern consistent with the sort of complex coevolutionary dynamics suggested by Alonzo (2010).

Second, experimental evolution can be used to directly measure how traits expressed in different family members evolve in response to different mating systems or social environments. This approach has been used extensively in the study of sexual selection and sexual conflict in organisms like *Drosophila melanogaster*, where many studies have examined the consequences of sexual selection by removing it from populations via enforced monogamy (e.g., Cally, Stuart‐Fox, & Holman, 2019; Hollis, Fierst, & Houle, 2009; Hollis & Kawecki, 2014; Pitnick, Miller, Reagan, & Holland, 2001; Veltsos, Fang, Cossins, Snook, & Ritchie, 2017; Yun et al., 2019). Such studies have provided important insights into the ways in which sexual selection and sexual conflict impact phenotypic evolution and population fitness. However, few studies have applied experimental evolution to the study of parental care (although see Head et al., 2014; Jarrett et al., 2018; Jarrett, Schrader, Rebar, Houslay, & Kilner, 2017; Kölliker et al., 2015; Schrader, Jarrett, & Kilner, 2015; Schrader, Jarrett, Rebar, Kilner, & Schrader, 2017). This is in part because many organisms with complex parental care behaviors have relatively long generation times making them unsuitable for experimental evolution.

**FIGURE 1:**
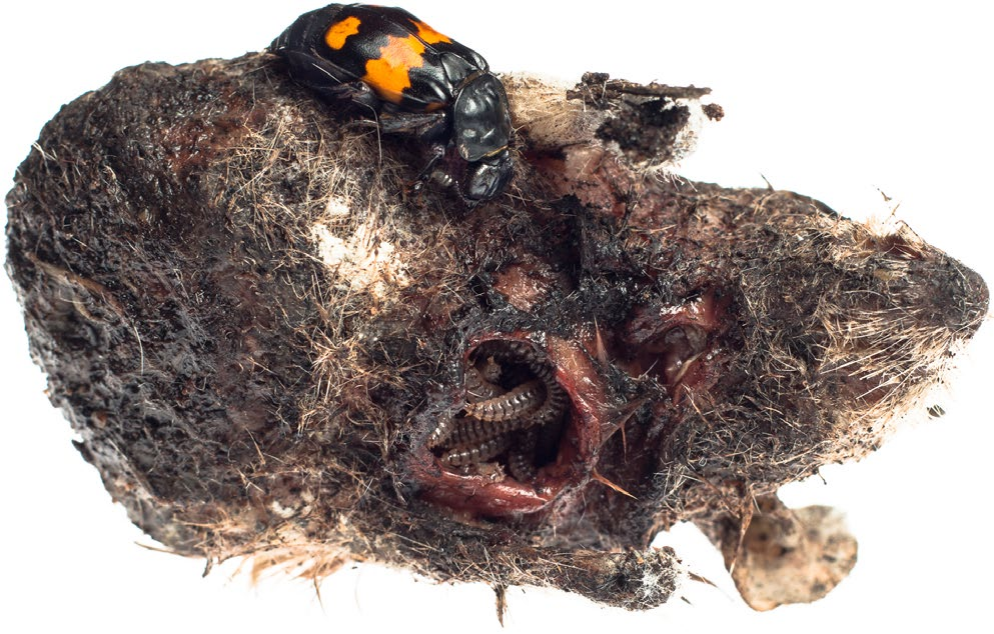
*Nicrophorus vespilloides* rearing a brood on a prepared mouse carcass. Photo by Tom Houslay

The burying beetle, *N. vespilloides* (Figure 1), displays complex parental care behaviors and has a generation time of ~30 days, which makes artificial selection and experimental evolution feasible. Recent studies of *N. vespilloides* have employed artificial selection and experimental evolution to examine how interactions among family members influence the evolution of mating and parenting behaviors (Head et al., 2014; Jarrett et al., 2017, 2018; Schrader et al., 2015, 2017). However, no studies have directly manipulated the mating system and measured the evolutionary consequences. Here we describe an experiment in which we manipulated the opportunity for promiscuity in experimental populations of *N. vespilloides* and measured the consequences for the evolution of mating and parenting behaviors. We first describe how our manipulation influences measures of reproductive success (egg production, brood size at dispersal, mean larval mass) and adult survival within a single generation. We then test for divergence in mating and parenting behaviors in populations that have been subjected to 6 generations of experimental evolution under different levels of reproductive competition. We specifically focus on the hypotheses that the males evolving in the promiscuous selection regime will exhibit higher mating rates and lower levels of parental care than beetles evolving under the monogamous selection regime.

## 2 METHODS

### 2.1 Study species

*Nicrophorus vespilloides* requires vertebrate carrion to breed (Figure 1), and there can be strong intrasexual competition for access to suitable carrion involving fights to establish a dominant role on the breeding carcass (Bartlett & Ashworth, 1988; Eggert & Müller, 2000; Eggert, Otte, & Müller, 2008; Otronen, 1988). The winners of these competitions remain to become the dominant carcass‐holding individuals while the losers may remain near the carcass as satellite individuals. Satellite males may attempt to mate with the dominant female and satellite females may act as brood parasites by laying eggs in the soil surrounding the carcass (Eggert et al., 2008; Müller, Braunisch, Hwang, & Eggert, 2007; Scott, 1998). The presence of satellite individuals has the overall effect of lowering the certainty of paternity and maternity of the dominant carcass‐holding male and female.

Reproduction in *N. vespilloides* also involves elaborate pre‐ and posthatching parental care (reviewed in Royle, Hopwood, & Head, 2013; Scott, 1998). Prehatching care lasts approximately 3 days and involves shaving the carcass, rolling it into a ball and burying it, and coating the surface with anti‐microbial exudates. Posthatching care involves feeding the larvae from the carcass, maintaining the carcass, and defending the carcass and brood from competitors. One of the more unusual aspects of parental care in *N. vespilloides* is that it can involve one (either male or female) or both parents and that the sexes differ in the level of care that they provide to larvae when they are caring alone versus when they are caring with the other parent. Specifically, males reduce the amount of care they provide to the brood in response to the presence of a caring female whereas females provide nearly the same amount of care whether they are providing care alone or with a male (Parker et al., 2015; Royle, Russell, & Wilson, 2014).

### 2.2 Experimental populations

The populations used in this study were derived from a laboratory‐adapted population of *N. vespilloides* that had been maintained at the University of the South, Sewanee, TN for 19 generations prior to the experiment. This population was originally founded with the laboratory‐born offspring of beetles collected from two localities in Cambridgshire, UK. Each generation, we bred 40–50 pairs of beetles. Mating was monogamous and random with the exception that we did not breed siblings. In the generation prior to the start of the experiment described below, the stock population was interbred with the laboratory‐born offspring of wild beetles collected from four locations in Cambridgeshire, UK. After interbreeding the lab population with wild beetles, we split it into the populations described below.

We maintained a total of four populations under two different selection regimes. Two populations were maintained under monogamy (M populations) and two under conditions that allow for promiscuity (P populations). For logistical purposes, pairs of populations were maintained 1 week out of sync with one another (i.e., M1 and P1 were bred one week and M2 and P2 were bred the next week). In the M populations, we randomly paired male and female beetles (excluding siblings), provided them with a thawed and weighed mouse carcass (13–18 g; RodentPro, Inglefield, IN), and allowed them to breed in a plastic box (box dimensions, length x width x depth: 15 cm × 13.4 cm × 8 cm) filled with ~2 cm of damp soil (Garden Magic Topsoil, Michigan Peat Company). These conditions eliminate intrasexual competition and maximize certainty of parentage. We bred beetles in the P populations in the same way, except that each breeding box contained 2 males and 2 females that were added to the breeding box simultaneously. Under these conditions, there is the potential for intrasexual competition, and satellite adults can reduce the parentage of the dominant female and male through brood parasitism and multiple mating, respectively.

For each breeding pair/quartet, we counted and weighed the dispersing larvae 8 days after pairing and placed each larva in a cell (cell dimensions: 2 cm × 2 cm × 1.8 cm) within a 25 cell “eclosion box” (box dimensions: 10 cm × 10 cm × 1.8 cm). All cells were covered with damp soil and the box was covered with a plastic lid. Upon eclosion, adults were placed in a plastic box (box dimensions, length × width × depth: 8.5 cm × 8.5 cm × 4 cm) containing a layer of damp soil (Garden Magic Topsoil) and a small amount of ground beef. Adult beetles were fed ground beef twice per week and were bred 14 days after eclosion as described above. Breeding pairs (or quartets in the P populations) were assigned randomly with the exception that siblings were not allowed to breed. Each generation, we bred 30–50 pairs in each of the M populations (mean = 42.25 pairs per generation) and 25–45 quartets in each of the P populations (mean = 37.33 quartets per generation).

### 2.3 The immediate impact of the mating system manipulation

We first examined how our different selection regimes influenced three measures of breeding success: egg production, brood size at dispersal, and mean larval mass at dispersal. Each of these measures was made in the first generation of the experiment, before any divergence was possible. Burying beetle eggs are laid over a period of 24–60 hr and hatch asynchronously (Smiseth, Ward, & Moore, 2006). This creates problems when trying to measure the total number of eggs that are laid in a clutch, because the earliest laid eggs will hatch before the last eggs are laid. To account for this, we used a protocol similar to that described by Smiseth et al. (2006). We began by breeding beetles under monogamy or promiscuity (as described above). Forty‐eight hours after setting up the breeding boxes (before the first laid egg hatched), we transferred the adults and the carcass to a new breeding box filled with damp soil. We then counted the number of eggs in the original box. This process was repeated once more, 24 hr after the adults had been transferred to the new breeding box. Egg production for each replicate was measured as the total number of eggs produced over these two time periods. The distribution of egg counts was skewed (in different directions) in each treatment. We thus compared the number of eggs produced in the two selection regimes using a nonparametric test (Wilcoxon test).

In a subsample (*n* = 19) of the promiscuous replicates described above, we used a dye‐marking technique (Eggert & Müller, 2000) to examine whether egg production was dominated by a single female. These replicates contained one female that had been fed normal ground beef for 2 weeks prior to breeding and one female that had been fed ground beef stained with Sudan Red 7B (200 mg of dye per 20 g of beef) for 2 weeks prior to breeding. Previous studies have shown that feeding females beef stained with Sudan Red results in the production of eggs that are stained red to pink, but does not affect the timing of oviposition, the number of eggs laid, hatching success, or larval survival (Eggert & Müller, 2000). The egg production of each female was measured by counting the number of red and white eggs produced in each box. We then quantified “clutch sharing” using the brood sharing metric described by Eggert and Muller (Eggert & Müller, 2000). This metric is calculated as 4*P_1_ *P_2_, where *P*_1_ and *P*_2_ are the proportion of the eggs laid by female 1 and female 2, respectively. This index varies between 1 (where both females contribute equally to the clutch) and 0 (where only one female lays eggs).

Brood size and mean larval mass were measured in each replicate population in the first generation of the experiment. Some previous studies have found that carcass mass influences brood size at dispersal and mean larval mass (Smiseth, Andrews, Mattey, & Mooney, 2014); however, we used a narrow range of carcass sizes in this experiment (mean = 15.86 grams, standard deviation = 1.52) and preliminary analyses of our data showed no relationship between carcass mass and these variables (regression of brood size on carcass mass, *F*_1,115_ = 0.61, *p* = .44; regression of mean larval mass on carcass mass, *F*_1,115_ = 1.28, *p* = .26). Thus, we did not include carcass mass in subsequent analyses.

We tested whether mean brood size at dispersal differed between the selection regimes using a *t* test. Mean larval mass was negatively correlated with brood size at dispersal (*r *= −.32, *p* < .0001, *n* = 115) so we tested whether the selection regime influenced mean larval mass using an ANCOVA with selection regime (monogamy or promiscuity) as the factor and brood size as the covariate. We initially included the treatment by covariate interaction in the model to test the homogeneity of slopes assumption. This interaction was not significant so it was removed from the model.

### 2.4 The impact of the selection regime on adult survival

During the first generation of the experiment we noticed adult mortality in many of the promiscuous replicates. Thus, in the second generation of the experiment, we measured the survival of breeding adults in each of the experimental populations. To do this, we bred beetles as described above and simply counted the number of beetles of each sex that were alive in each breeding box at larval dispersal (8 days after pairing). We first used a *χ* ^2^ test to test whether the number of breeding attempts in which there was adult mortality (of either sex) was independent of the selection regime. We next tested whether patterns of mortality were independent of sex in the promiscuous populations. We focused on sex‐differences in survival in the promiscuous populations because mortality was very low in the monogamous populations (see results below). Note that our methods allowed us to measure and compare mortality between the sexes, but did not allow us to track the survival of individual beetles.

### 2.5 Mating behaviors

After six generations, we used scan sampling of experimental pairs to measure male mating behaviors in each experimental population. The experimental pairs involved 14–16 day‐old virgins in all possible combinations of male and female selection regime within each set of replicate populations (e.g., M1_female_ × M1_male_, M1_female_ × P1_male_, P1_female_ × M1_male_, P1_female_ × P1_male_). By conducting crosses within and between populations, we are able to test whether mating behavior is due to the male population of origin, the female population of origin, or the interaction between the two (as in Head et al., 2014). We did not conduct crosses between sets of replicate populations (e.g., M1_female_ × M2_male_) because the two sets of replicate populations were maintained 1 week apart. Thus, these crosses would involve individuals that varied systematically in age. Cross types were replicated between 9 and 15 times. We put each experimental pair in a small plastic container covered with a transparent lid (box dimensions, length × width × depth: 8.5 cm × 8.5 cm × 4 cm). Each pair was scan‐sampled 30 times (once per minute) and at each scan we recorded the following behaviors: mating (with or without female resistance), mate guarding (with or without resistance), chasing, or beetles apart. Mating occurred whenever the male was observed to have inserted his aedeagus into the female. We considered mating to occur without resistance if the female was still during mating. If the female was moving or trying to dislodge the male, then we considered mating to occur with female resistance. Mate guarding was defined as occurring when the male was within a pronotum width of the female but was not mating. Mate guarding often involved the male remaining on top of the female, but without his abdomen curled under hers. We considered mate guarding to occur without resistance if the female was still while the male was guarding. If the female was moving or trying to dislodge the male, then we considered mate guarding to occur with female resistance. The beetles were considered to be apart if they were >1 body length apart. All pairs were observed blindly with respect to cross type.

We used principal component analysis (PCA) to create composite variables summarizing male mating behaviors (Head et al., 2014). This analysis resulted in two principal components (PCs) that together explained 56% of the variation in male mating behavior. PC1 captured 33% of the variation in the mating behaviors. Mate guarding without resistance and mating without resistance loaded positively on this axis (0.84 and 0.60, respectively) and being apart loaded negatively (−0.94). PC2 explained 23% of the variation in male mating behavior. Mate guarding with resistance and mating with resistance loaded positively on this axis (loadings were 0.84 and 0.70) and mate guarding without resistance loaded negatively (−0.39).

We extracted PC scores from the principal components analysis and then compared them between populations using factorial ANOVAs (one for each PC) to test whether the male selection regime, female selection regime, and their interaction determined male mating behavior (summarized as PC1 and PC2). In these analyses, we initially included the replicate population as a fixed effect. This was done to account for potential variation among replicate populations due to either idiosyncratic evolutionary changes or the fact that the populations were temporally staggered (as in Schrader et al., 2015). Replicate population and interactions involving replicate population were not significant and were removed from the final model.

### 2.6 Parenting behavior and breeding success

After six generations, we measured parental attendance in beetles descended from the monogamous and promiscuous populations. Within each population, we paired unrelated males and females and bred each pair monogamously as described above. At 53 hr after pairing, we assigned each of the breeding pairs to one of two groups: female‐only care or male‐only care and then removed the appropriate parent. This was done because previous studies, (and a preliminary experiment in our populations) have shown that males provide very little parental care when they are caring with a female, whereas female care is relatively insensitive to the presence of a male (Parker et al., 2015; Royle et al., 2014). We began with 15 replicates of each treatment per replicate population. However, the sample size for our measurement of parental attendance was reduced by hatching failures, adult death, and disappearance of larvae from the carcass. The final sample size for each treatment was: M1_female care_ = 9, M1_male care_ = 10, M2_female care_ = 15, M2_male care_ = 11, P1_female care_ = 9, P1_male care_ = 10, P2_female care_ = 11, P2_male care_ = 13.

The duration of parental care was measured by observing each breeding box 15 times beginning 72 hr after pairing. Observations were made at 0700, 1100, 1500, and 1900 each day except on the last day when we observed the boxes at 0700, 1100, and 1500. During each observation we recorded whether a parent was with the brood. We considered the parent to be present if it was on the carcass and at least one larva was present; however, we did not attempt to measure what type of care the parent was engaged in during the observation. For each replicate, parental attendance was calculated as the total number of observation periods during which the parent was observed to be with the brood. Eight days after pairing, we counted the number of dispersing larvae in each breeding box. Observations of parental attendance and number of dispersing larvae were made blindly with respect to the selection regime.

We used a general linear model (quasi‐Poisson error and log link) to examine the impact of the selection regime (monogamous or promiscuous), parental sex (female or male), the replicate population (to account for effects of drift, and/or staggering the populations), and their interactions on parental attendance. Nonsignificant terms (*p* > .05) were removed from the model beginning with the three‐way interaction. We used the same approach to test for effects of the selection regime, parental sex, replicate population, and their interactions on brood size at dispersal. All analyses were performed in R (R Development Core Team, 2016).

## 3 RESULTS

**FIGURE 2:**
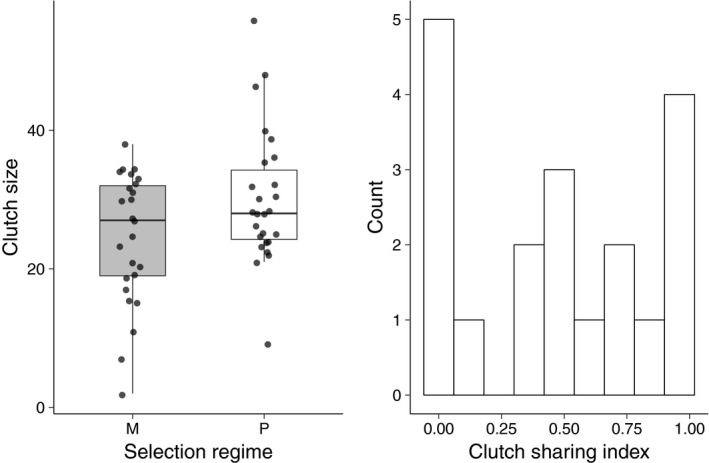
The impact of the selection regime (monogamy or promiscuity) on clutch size (a) and the distribution of clutch sharing (b) under promiscuity. Clutch sharing was calculated at *P*1*P2*4 where *P*1 and *P*2 are the proportion of eggs within the clutch attributed to female 1 and female 2, respectively. Values of 0 correspond to a situation where one female lays all of the eggs in a clutch and values of 1 correspond to a situation where the clutch is split evenly between the two females

**FIGURE 3:**
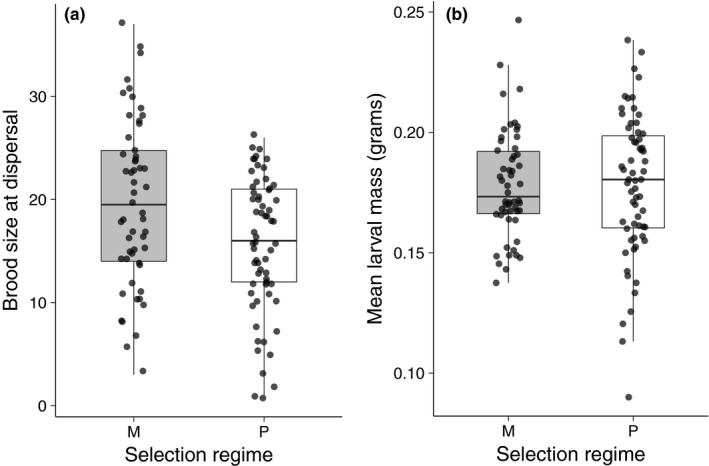
The impact of the selection regime, monogamy (M) or promiscuity (P), on (a) brood size at dispersal and (b) mean larval mass at dispersal

The distribution of egg production was skewed toward small clutches under monogamy and large clutches under promiscuity (Figure 2a); however, there was no significant difference between these two treatments in egg production (Wilcoxon rank sum test, *p* = .13). In the 19 replicates in which we measured clutch sharing, 14 contained eggs laid by both females. The distribution of clutch sharing values indicated that it was just as common for one female to exclude the other as it was for both females to contribute to the clutch equally (Figure 2b). Although the mean clutch size was about 5 eggs larger under promiscuity than monogamy, brood size at dispersal was significantly smaller in the promiscuous populations than in the monogamous populations (*t* = 3.05; *p* = .0029), with promiscuous broods containing on average 4 fewer larvae at dispersal than monogamous broods (Figure 3a). Mean larval mass was negatively affected by brood size at dispersal (ANCOVA, *F*_1,114_ = 14.48, *p* = .00023), however there was no difference between the selection regimes in mean larval mass (ANCOVA, *F*_1,114_ *= *0.0, *p* = .98; Figure 3b).

Patterns of adult survival differed between the selection regimes, with adult survival during breeding being lower under promiscuity than monogamy (proportion of monogamous breeding attempts with adult mortality: 0.08, *n* = 100; proportion of promiscuous breeding attempts with adult mortality: 0.82, *n* = 78; *χ* ^2^ = 67.3, *p* < .00001). Within the promiscuous populations, we further tested whether mortality during breeding was independent of sex by comparing the frequency of broods with 0, 1, or 2 surviving adults between the two sexes. We found that the frequency of broods with 0, 1, or 2 surviving adults was not independent of sex (*χ* ^2^ = 8.99, *p* = .01; Table 1), with males having higher mortality than females during breeding under promiscuous conditions (Table 1).

**TABLE 1:** Patterns of male and female survival during breeding under promiscuity

Sex	Number of surviving adults		
	0	1	2
Female	2	32	44
Male	3	49	26

Note

For each sex, we list the number of breeding quartets in which 0, 1, or 2 adults survived until larval dispersal.

### 3.1 Mating behaviors

**FIGURE 4:**
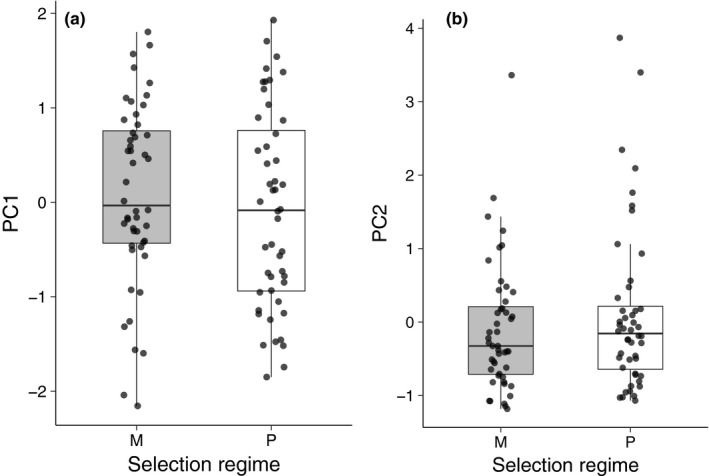
A comparison of male mating behaviors between males from the monogamous (M) and promiscuous (P) selection regimes. A principal components analysis was used to create composite measures of male mating behavior. PC1 (a) captured 33% of the variation in the mating behaviors. Mate guarding without resistance and mating without resistance loaded positively on this axis (0.84 and 0.60, respectively) and being apart loaded negatively (−0.94). PC2 (b) explained 23% of the variation in male mating behavior. Mate guarding with resistance and mating with resistance loaded positively on this axis (loadings were 0.84 and 0.70) and mate guarding without resistance loaded negatively (−0.39). For illustrative purposes, crosses involving males from the same selection regime are pooled

Mating behavior as summarized by PC1 was not affected by the selection regime of the male population (*F*_1,91_ = 0.70, *p* = .404; Figure 4a), the selection regime of the female population (*F*_1,91_ = 0.30, *p* = .58), or the interaction between the two (*F*_1,91_ = 1.7, *p* = .19). Similarly, PC2 was not affected by male selection regime (*F*_1,91_ = 1.01, *p* = .32; Figure 4b), the female selection regime (*F*_1,91_ = 0.02, *p* = .89), or the interaction between the two (*F*_1,91_ = 0.24, *p* = .63). Together these results indicate that there has been no divergence between the monogamous and promiscuous populations in the mating behaviors that we measured.

### 3.2 Parental attendance and breeding success

**FIGURE 5:**
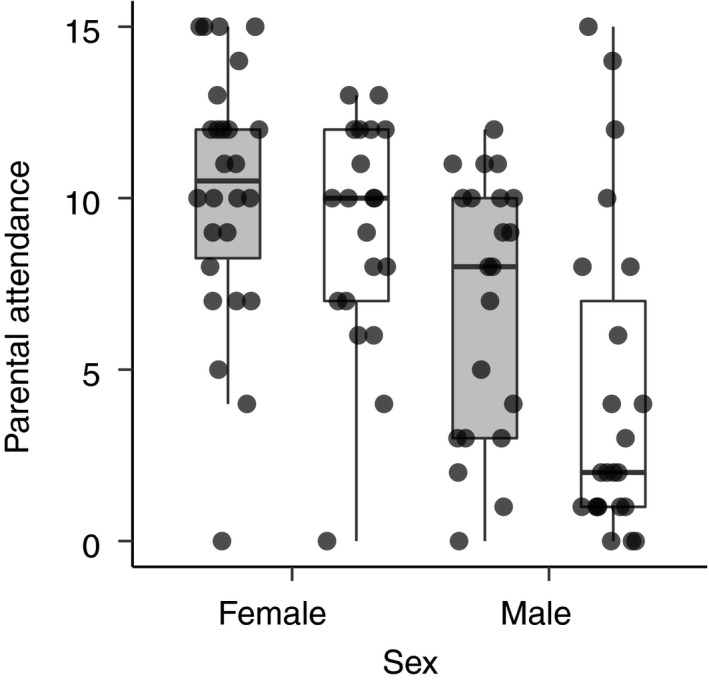
Parental attendance of females and males from the monogamous (gray boxes) and promiscuous (white boxes) populations. Parental attendance was measured as the number of observation periods during which the parent was observed to be with the brood

The reduced model for parental attendance included significant effects of sex (*F*_1,87_ = 19.56, *p* < .0001) and the selection regime (*F*_1,87_ = 4.33, *p* = .040). Females exhibited greater attendance than males and parental attendance was higher in the monogamous populations than in the promiscuous populations (Figure 5). This difference appeared greatest in males (Figure 5).

**FIGURE 6:**
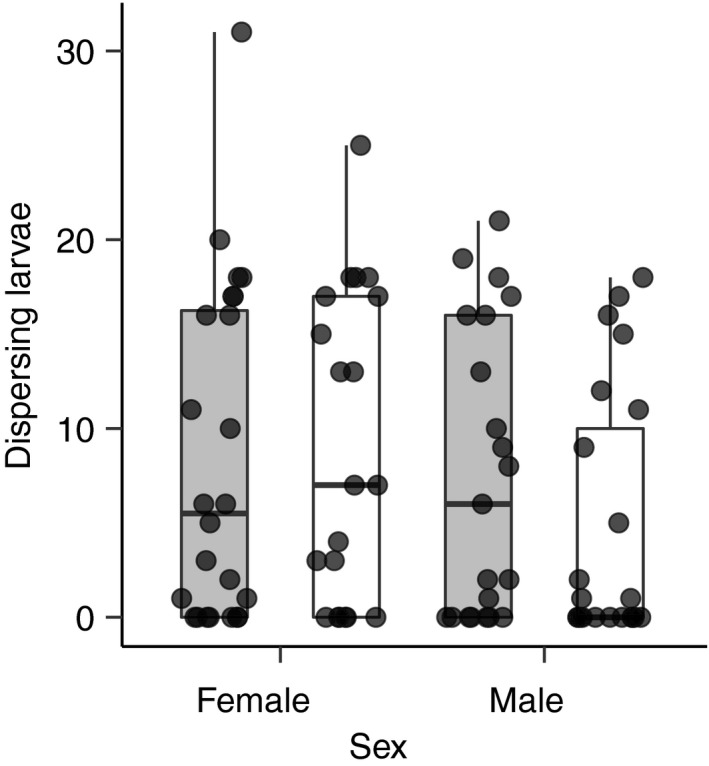
The number of dispersing larvae in broods cared for by monogamous (gray boxes) and promiscuous (white boxes) females and males

We only measured parental attendance in replicates where larvae were observed on the carcass. However, several of these replicates failed to produce any dispersing larvae during the experiment. Although these failures were most common when P males were providing care (Figure 6) we found no significant effects of sex (*F*_1,89_ = 2.58, *p* = .11) or the selection regime on brood size at dispersal (*F*_1,89_ = 0.53 *p* = .47).

## 4 DISCUSSION

Experimental evolution has been used extensively to examine how populations evolve in response to changes in the mating system. However, these studies have focused almost entirely on how mating behaviors evolve in response to different levels of sexual selection. We know much less about how adaptation to different mating systems might impact the coevolution of mating and parenting behaviors (Alonzo, 2010; Royle et al., 2016). In this study we examined the utility of experimental evolution for studying the evolution of mating and parenting behaviors. Our first goal was to examine the immediate impact of a simple mating system manipulation on measures of reproductive performance and adult survival. Our second goal was to test whether adaptation to different mating systems involves concurrent changes in mating and parenting behaviors.

### 4.1 The impact of the mating system on reproductive performance and adult survival

Our manipulation of the mating system had mixed effects on measures of reproductive performance and adult survival. We found that clutch size was not significantly different between the two selection regimes (Figure 2a), despite evidence from a subset of the promiscuous replicates indicating that both females frequently contributed to the clutch (Figure 2b). The fact that clutch size did not differ between the two selection regimes suggests that egg production may be constrained by carcass size even when two competing females contribute to egg production. Our results also suggest that in the promiscuous selection regime, caring females may often rear larvae that are not their genetic offspring. This type of brood parasitism is probably common in nature (Eggert et al., 2008; Müller et al., 2007; Scott, 1998) and it appears that our manipulation of the mating system in the lab can generate the same phenomenon.

Although clutch size did not differ between the two selection regimes, brood size at dispersal was significantly lower under promiscuity than monogamy (Figure 3). This result suggests that there was greater brood reduction in the promiscuous selection regime than the monogamous selection regime. The mechanism behind the apparent brood reduction in our experiment is unclear. However, Eggert et al. (2008) have suggest that increased brood reduction under reproductive competition may be a consequence of infanticide by the dominant female or male. Previous studies have shown that burying beetle parents use temporal cues generated by hatching asynchrony to cull larvae, possibly as a defense against brood parasitism (Eggert et al., 2008; Muller & Eggert, 1990; Trumbo, 1990). Thus, greater hatching asynchrony under promiscuity may be the proximate mechanism generating the differences in brood reduction that we observed here. It is also possible that the brood reduction we observed in the promiscuous selection regime was driven by interactions among adult beetles. For example, male–male and female–female aggression were possible in the promiscuous selection regime and these interactions may have reduced the amount of time parents spent caring for larvae. Similarly, increased mating opportunities may have resulted in a reduction in the amount of parental care. Studies of birds have revealed such trade‐offs (e.g., Duckworth, 2006), however assessing these possibilities in our populations will require more detailed observations of aggression, mating, and parenting under the promiscuous selection regime.

Although there were not large differences between the two selection regimes in measures of breeding performance, there were considerable differences in adult mortality. Under monogamy, adult mortality during breeding was quite low (there was mortality of one or more adults in only 8% of the monogamous pairs), whereas under promiscuity adult mortality was 10 times higher (there was mortality of one or more adults in 82% of the promiscuous quartets). Furthermore, mortality in the promiscuous populations was considerably higher for males than females (see Table 1). Although we did not measure male–male aggression in our experiment, many of the dead adults showed signs of having been involved in fights (e.g., missing legs, missing heads, torn abdomens).

The high male mortality in the promiscuous selection regime suggests that reproductive competition might generate strong selection on males. We did not individually mark and measure the beetles in our experiment so we are not able to test whether male mortality was selective. However, the outcome of intrasexual aggression in *N. vespilloides* is often size‐based with larger individuals winning (Bartlett & Ashworth, 1988). Thus, selection in our promiscuous populations generated by male–male combat may favor larger males. Whether this type of selection will lead to an evolutionary increase in male size is unclear as the heritability of body size in *N. vespilloides* is low (Jarrett et al., 2017). It is also possible that selection in the promiscuous populations favors males that show higher levels of aggression toward competitors independent of body size, and that this selection is relaxed under monogamy. Future studies observing intrasexual interactions between marked individuals will be useful for determining whether adult mortality is selective.

### 4.2 Divergence in mating and parenting behaviors

Our second major goal was to test whether adaptation to different mating systems involved the (co)evolution of mating and parenting behaviors. Previous studies suggest that sperm competition in *N. vespilloides* may favor increased male mating rate (House, Hunt, & Moore, 2007; Muller & Eggert, 1989) and artificial selection on male mating rate results in a correlated decline in female (but not male) parental care (Head et al., 2014). Thus, adaptation to promiscuity might entail an increase in male mating rate and a corresponding decrease in female care. Our results are not consistent with these predictions. First, we found no evidence that adaptation to the different selection regimes used in this study involved evolutionary changes in male mating behaviors. Second, our results suggest that adaptation to different mating systems involved a consistent change in the duration of parental care, with parents (especially males) from the monogamous populations displaying greater parental attendance than males from the promiscuous populations.

It is possible the differences between our results and those of Head et al. (2014) are a consequence of the different approaches employed. Specifically, Head et al. (2014) used artificial selection on mating rate while we did not select on a specific trait, but instead manipulated the potential for promiscuity across populations. These two approaches both have merit but offer different insights. For example, Head et al.' s (2014) approach offers a direct test of whether mating behaviors and parenting behaviors are genetically correlated with one another in *N. vespilloides*. However, artificial selection on mating rate may not capture the complexity of selection in social environments where males have to compete with one another directly via intrasexual aggression, as well as via sperm competition. Indeed, the high mortality that we observed suggests that male fitness in our promiscuous populations is determined more by the outcome of male–male combat than sperm competition. The experimental evolution approach we employed allows us to examine adaptation to potentially complex social environments. However, without more detailed measurements of individual interactions, our approach does not allow us to quantify the importance of direct and indirect selection generated by these social environments.

The observation that males from the monogamous populations displayed greater parental attendance is consistent with the hypothesis that male care will increase with the certainty of paternity. However, it appears that this change is not associated with a concurrent change in mating behavior. Although this suggests that the evolution of male care and mating behaviors are not linked to one another, it is possible that the evolution of increased male attendance in the monogamous populations is associated with changes in other behaviors that we have yet to measure. For example, the importance of intrasexual aggression differs between the two selection regimes in our experiment. If there is a trade‐off between aggression and male parental care, then relaxed selection for aggression in the monogamous populations could result in a correlated increase in male parental care. We are unaware of studies that have tested for a trade‐off between aggression and paternal care in *N. vespilloides*. However, a phenotypic trade‐off between aggression and parenting has been demonstrated in several species of birds in which males have to balance investment in territory defense and investment in parental care (Duckworth, 2006; McGlothlin et al., 2007). Future studies examining the potential links between intrasexual aggression and parental care behaviors in *N. vespilloides* might help explain some of our results.

We found no significant differences between sexes or populations in the number of dispersing larvae. However, a large number of the broods cared for by promiscuous males failed to produce any dispersing larvae (Figure 6). Because we did not control for the number and origin of larvae in the broods that we observed, we cannot assess whether reductions in male care are a cause or consequence of poor larval performance. Previous studies (e.g., Head et al., 2014) have successfully manipulated the number and origin of larvae to control for brood size and relatedness. We attempted to use the same techniques in a pilot study. However, larval mortality was very high between hatching and assignment of larvae to experimental broods. Thus, we decided to conduct our experiment using unmanipulated broods. Future manipulations of brood size and larval origin will be necessary to test whether promiscuous males provide less care than monogamous males independent of any effects of brood size on the duration of care.

Finally, we note that our conclusions regarding divergence (or lack thereof) in mating and parenting behaviors only apply to the social environments in which they were measured. Mating behavior was only assayed under monogamy and it is possible that differences between selection regimes in male mating behavior are only expressed under promiscuity (i.e., when there are two males and two females present). Similarly, we only assayed parental care under monogamy and uniparental care. Thus, it is possible that differences between selection regimes in parental care are sensitive to the mating environment (e.g., whether mating was monogamous or promiscuous) or the social environment during parental care (i.e., whether care is uniparental or biparental). We hope to examine these possibilities in future studies.

### 4.3 Future directions

Our manipulation of the mating system was designed to create extreme differences between populations in certainty of paternity, however there are additional components of the mating system that we did not incorporate in our selection regimes that might be interesting to examine in future studies. First, in nature female *N. vespilloides* can mate away from a carcass and store sperm (Scott, 1998). This means that females attracted to a breeding carcass will often be carrying sperm and that the carcass‐tending male will almost always face sperm competition (even if there are no satellite adults present near the carcass). Second, the size of the breeding carcass has been shown to influence the tolerance of dominant adults to subordinate males and females. Specifically, dominant adults are more tolerant of subordinates when breeding on large carcasses than when breeding on small carcasses (Eggert et al., 2008). We used relatively small carcasses in this experiment which will increase the likelihood intrasexual aggression. Finally, in our experiment individuals were only given a single opportunity to breed thus there was no opportunity for a trade‐off between parental investment in the current brood and investment in future broods. Such a trade‐off is an important assumption of theory predicting that males should plastically adjust parental care in response to the mating system (Westneat & Sargent, 1996; Westneat & Sherman, 1993). It might be possible to incorporate some of these complexities into future studies.

## CONFLICT OF INTEREST

None declared.

## AUTHOR CONTRIBUTIONS

**Matthew Schrader:** Conceptualization (lead); data curation (lead); formal analysis (lead); funding acquisition (lead); investigation (lead); methodology (lead); project administration (lead); resources (lead); supervision (lead); writing–original draft (lead); writing–review and editing (lead). **Madolin K. Keller:** Conceptualization (supporting); data curation (equal); methodology (supporting); writing–original draft (supporting). **Garrett F. Lucey:** Conceptualization (supporting); data curation (supporting); investigation (supporting); methodology (supporting); writing–original draft (supporting).

## Data Availability

Data have been uploaded to DRYAD: https://doi.org/10.5061/dryad.ngf1vhhr0
